# Compartment-specific skeletal involvement in systemic sclerosis characterized by peripheral QCT and serum osteoimmune mediators: a case-control study

**DOI:** 10.1007/s00223-026-01624-5

**Published:** 2026-10-22

**Authors:** Dóra Csige, Ágnes Horváth, Monika Bodoki, István Rebenku, Sára Lipták-Lukácsik, Lilla Ramóna Pomázi, Zsófia Pethő, Sándor Mogyoróssy, Attila Hamar, Nóra Bodnár, Rebeka Falcsik, Katalin Hodosi, György Panyi, Szilvia Szamosi, Zoltán Szekanecz, Gabriella Szűcs, Edit Végh, Levente Bodoki

**Affiliations:** 1 Division of Rheumatology, Department of Internal Medicine, Faculty of Medicine, University of Debrecen, Móricz Zsigmond út 22, Debrecen, 4032 Hungary; 2 Doctoral School of Clinical Medicine, University of Debrecen, Debrecen, Hungary; 3 Department of Biophysics and Cell Biology, Faculty of Medicine, University of Debrecen, Debrecen, Hungary

**Keywords:** Systemic sclerosis, pQCT, Bone markers, Osteoimmunology

## Abstract

Systemic sclerosis (SSc) is associated with increased bone fragility, yet early, compartment-specific alterations and their osteoimmunological correlates remain incompletely characterized. This study aimed to assess bone status in SSc using dual X-ray absorptiometry (DXA) complemented by peripheral quantitative computed tomography (pQCT), and to evaluate their associations with a targeted osteoimmunological biomarker panel and clinical risk scores. This prospective cross-sectional study included 40 women with SSc (disease duration ≤ 7 years from diagnosis) and 45 non-immune female controls, frequency-matched by age. Bone status was assessed by DXA (lumbar spine, femoral neck) and distal radius pQCT. Serum levels of routine bone and mineral metabolism markers, alongside a targeted panel of osteoimmunological mediators (OPG, OPN, PDGF-BB, ACP5, RANKL, TNF-α, IL-6, IL-1β, DKK-1) using multiplex immunoassay were measured. FRAX scores were calculated to contextualize clinical fracture risk. With broadly comparable DXA-defined bone status, pQCT revealed significantly lower trabecular volumetric bone mineral density (vBMD) in SSc, while total and cortical+subcortical vBMD were comparable. Routine biochemical markers showed substantial overlap between groups, whereas SSc patients exhibited a distinct osteoimmune profile with significantly elevated OPG, ACP5, RANKL, PDGF-BB, IL-6, IL-1β, DKK-1, and RANKL/OPG after FDR correction. Exploratory biomarker–bone associations did not remain significant after correction for multiple testing. In women with relatively early SSc, pQCT identified compartment-specific differences, particularly within the trabecular compartment, despite comparable DXA-defined bone status. A distinct osteoimmunological biomarker profile was also observed in SSc, providing complementary information on disease-related skeletal alterations, while specific biomarker–bone associations require further confirmation.

**Supplementary Information:**

The online version contains supplementary material available at https://doi.org/10.1007/s00223-026-01624-5.

## Introduction

SSc is a rare chronic autoimmune connective tissue disease characterized by immune dysregulation, microvascular injury, and progressive fibrosis, leading to multi-organ involvement and increased morbidity. Emerging data indicate that SSc is associated with impaired skeletal health, including reduced BMD and an increased risk of fragility fractures compared with the general population, even after adjustment for traditional osteoporosis risk factors such as age, menopausal status, and body mass index (BMI) [1–3]. However, reported effect sizes and the relative contribution of disease-specific mechanisms remain heterogeneous across studies, suggesting that SSc-related factors may contribute to bone fragility beyond classical determinants [4, 5].

BMD assessment in routine clinical practice relies primarily on DXA, which provides areal BMD (aBMD) measurements at clinically relevant skeletal sites and forms the basis of osteoporosis diagnosis and fracture risk prediction tools such as the Fracture Risk Assessment Tool (FRAX) [6, 7]. While DXA is indispensable for clinical decision-making, it represents a two-dimensional projection of bone mineral content and does not allow differentiation between trabecular and cortical compartments. These limitations may be particularly relevant in systemic diseases, where compartment-specific alterations may influence bone strength and fracture susceptibility without necessarily resulting in marked reductions in aBMD [5, 8–10].

Three-dimensional volumetric BMD (vBMD) assessment with separate quantification of trabecular and cortical bone compartments is provided by pQCT, offering complementary structural information beyond DXA [10–11]. Measurements obtained at peripheral skeletal sites, such as the distal radius, have been shown to correlate with central DXA-derived BMD at the spine and hip, supporting their relevance as indicators of systemic skeletal integrity [12, 13]. Importantly, pQCT may add relevant structural detail regarding compartment-specific skeletal changes that are not fully reflected by aBMD, particularly in disease settings characterized by inflammatory and microvascular pathology [4, 10, 14]. Despite these advantages, the relationship between conventional DXA measures and compartment-specific pQCT parameters has not been thoroughly elucidated in SSc with short disease duration.

Fracture risk estimation commonly integrates clinical risk factors through the FRAX algorithm, which provides 10-year probabilities of major osteoporotic and hip fractures [6]. Although FRAX is widely used in clinical practice, its performance in inflammatory and autoimmune diseases may differ from that in the general population, as disease-specific factors such as chronic inflammation, vascular dysfunction, and altered bone remodeling are not explicitly incorporated [8, 9]. Accordingly, there is increasing interest in identifying imaging- and biomarker-based correlates that may complement DXA-derived BMD and FRAX-based risk assessment in SSc [15, 16].

Beyond imaging, biochemical markers of bone and mineral metabolism provide additional insight into skeletal dynamics. Routine laboratory parameters typically reflect preserved systemic mineral homeostasis and have limited sensitivity for detecting SSc-related skeletal alterations [17, 18]. In contrast, increasing attention has focused on osteoimmunological pathways linking immune activation and bone metabolism, including the RANKL-OPG axis, pro-inflammatory cytokines (e.g. IL-6, TNF-α, IL-1β), osteoclast-related enzymes (ACP5), and regulators of Wnt signaling such as Dickkopf-1 (DKK-1) [19–21]. These pathways are biologically plausible mediators of SSc-associated bone alterations. However, their relationships with imaging-derived bone parameters and clinical characteristics remain insufficiently explored in SSc, where complex alterations in tissue remodelling and vascular supply, as well as inflammation may modulate bone structure in ways that differ from typical osteoporotic populations.

Most previous studies investigating skeletal involvement focus on long-standing SSc, while patients with relatively short disease duration, defined here as ≤ 7 years since diagnosis, concordantly with widely used clinical trial definitions [22], represent a clinically informative population in which skeletal alterations can be examined with fewer cumulative confounders (e.g. prolonged, low-dose glucocorticoid exposure, or long-term immobility). Comparing this disease stage with non-immunological controls, may help disentangle disease-related skeletal effects from secondary consequences of chronic illness. This study aimed to explore associations between imaging-based bone parameters, routinely available laboratory markers, a targeted panel of bone-metabolism-related serum molecules, and clinical characteristics (including disease subtype, disease duration, BMI, and gastrointestinal involvement). Furthermore, we evaluated how these factors relate to FRAX-derived fracture risk to facilitate a direct comparison with the established risk prediction model, thereby contextualizing the clinical relevance of our molecular findings.

## Methods

### Study population

The study included 40 female patients with SSc and 45 female control subjects without immune-mediated disease. All participants were recruited and evaluated at a single center (Department of Rheumatology and Immunology, Clinical Centre, University of Debrecen, Hungary). SSc was diagnosed according to established EULAR/ACR classification criteria [23]. Disease duration was defined from the date of diagnosis, with inclusion restricted to patients with a maximum disease duration of ≤ 7 years. SSc subtypes were classified as limited cutaneous (lcSSc) or diffuse cutaneous (dcSSc) disease [24] (Table 1).

Control participants were frequency-matched to the SSc cohort by age. Osteopenia and osteoporosis were not exclusion criteria for controls, and during recruitment, representation across DXA-defined bone-status categories was balanced to allow comparisons between SSc and non-SSc participants with broadly comparable conventional skeletal status. This sampling strategy was intended to facilitate the assessment of compartment-specific pQCT and osteoimmune characteristics rather than to estimate differences in osteoporosis prevalence between SSc and the general population. Controls with a history of immune-mediated disease (including organ-specific autoimmune disorders, e.g. autoimmune thyroid disease) or current or previous malignancy were excluded.

No formal a priori sample size calculation was performed; the sample size was determined by the number of eligible participants recruited during the study period.

### Clinical characteristics

BMI was calculated as weight divided by height squared (kg/m^2^). Gastrointestinal involvement was assessed using the UCLA Scleroderma Clinical Trial Consortium Gastrointestinal Tract 2.0 (UCLA SCTC GIT 2.0) questionnaire, administered to both patients and controls and evaluated according to validated scoring guidelines [25].

### Dual-energy X-ray absorptiometry (DXA)

DXA was used to assess aBMD (GE Healthcare, Lunar Prodigy, Madison, Wisc., USA). Measurements were obtained at the lumbar spine (L1-4) and femoral neck (FN) according to standardized clinical protocols. All scans were performed by the same two experienced technicians, and routine quality control procedures were applied. BMD values were expressed in g/cm^2^, and T-scores were calculated using the manufacturer’s reference database [26]. Osteoporosis was defined as a L1-4 or FN BMD T-score ≤ − 2.5 SD, and osteopenia as a T-score between − 1.0 and − 2.5 SD, according to WHO criteria.

### Peripheral quantitative computed tomography (pQCT)

Peripheral volumetric bone measurements were obtained using a Stratec XCT-2000 L scanner (Stratec Medizintechnik GmbH, Pforzheim, Germany) at the distal epiphysis of the non-dominant radius. Slice positioning was guided by a scout scan to identify the distal radial endplate, which served as the anatomical reference point. Based on this reference, a standardized axial slice was acquired at 4% of the distal radius length, ensuring consistent positioning across patients. vBMD was quantified for total, trabecular, and cortical+subcortical compartments using a voxel size of 0.59 mm and analyzed with the manufacturer’s XCT software (version 6.00B; Stratec Medizintechnik GmbH). For image analysis, the measuring mask was set to the radius, with a density threshold of 269 mg/cm^3^, and the trabecular compartment was defined as the central 45% of the total bone cross-sectional area. During participant registration, the “Caucasian” ethnic group available in the XCT software was selected consistently for all participants. For pQCT-derived total and trabecular vBMD, T- and Z-scores were automatically generated by the XCT analysis software using the manufacturer-provided “German extended 960” reference dataset for the radius. The CALCBD reference function compares total and trabecular bone density with values from a healthy reference population matched for age and sex, with the reference values displayed in relation to the population mean and ± 1 and ± 2 standard deviations. Daily quality assurance procedures were performed using standard phantoms to ensure measurement stability. Missing pQCT data were attributable to unavailable scans, motion artefacts, or measurements not being performed.

### Fracture risk assessment (FRAX)

Ten-year probabilities of major osteoporotic fracture and hip fracture were calculated using the FRAX^®^ algorithm, a widely used fracture risk prediction model, configured for the Hungarian population, incorporating FN aBMD derived from DXA measurements.

Prevalent major osteoporotic fractures (MOF) were identified from the available clinical records and were defined as previous fragility fractures at major osteoporotic sites, including the hip, clinical vertebrae, proximal humerus, and distal forearm/wrist. Fractures at other sites (e.g., ankle, foot, toe, or hand) were not classified as MOF. In SSc patients with available pQCT measurements, pQCT-derived total, trabecular, and cortical+subcortical vBMD were compared between patients with and without prevalent MOF.

### Laboratory measurements

Routine laboratory parameters of bone and mineral metabolism were measured, including total calcium, phosphate, osteocalcin, bone-specific alkaline phosphatase, parathyroid hormone, 25-hydroxy vitamin D, vitamin B12, folate, and established bone turnover markers (CTX and P1NP).

Serum concentrations of bone-metabolism-related molecules were quantified using a multiplex bead-based immunoassay (Human Bone Metabolism Panel (13-plex), LEGENDplex™, BioLegend, San Diego, CA, USA), according to the manufacturer’s instructions. Venous blood samples were centrifuged at 4000 g, and the serum was separated from the cells. Serum samples were stored at − 80 °C until analysis. The analyzed analytes included markers of osteoclastogenesis, inflammation, and bone formation pathways (OPG, OPN, PDGF-BB, ACP5, RANKL, TNF-α, IL-6, IL-1β, DKK-1). Two serum samples of the SSc group were excluded from the final analyses due to gross lipemia, which can interfere with immunoassay performance and compromise measurement reliability [27]. This decision was consistent with the assay manufacturer’s sample handling recommendations for high-lipid specimens [https://www.biolegend.com/]. All samples were analysed in duplicate. Measurements were performed across assay plates under standardized experimental conditions. SSc and control samples were distributed across all plates in approximately balanced proportions to reduce the potential for systematic plate-related bias. According to the manufacturer’s validation data, intra-assay coefficients of variation for the biomarkers included in the present analysis ranged from 5 to 12%, while inter-assay coefficients of variation ranged from 6 to 23%, depending on the analyte and concentration tested.

### Statistical analysis

Statistical analyses were conducted using the SPSS Statistics software, version 29.0 (IBM Corp., Armonk, NY, USA). Data distribution was assessed for normality using the Shapiro-Wilk test and by visual inspection of histograms and Q-Q plots. Continuous variables are presented as mean (± standard deviation (SD)) or median (interquartile range) depending on data distribution, and categorical variables as counts (percentages). Student’s t-test or Mann–Whitney U test and Fisher’s exact test or chi-square test were used for between-group comparisons, as appropriate. Correlations were assessed using Pearson’s or Spearman’s correlation coefficients depending on data distribution. Univariable and multivariable linear regression analysis were used to assess associations between molecular markers and bone parameters. DXA- and pQCT-derived BMD parameters were treated as dependent variables, molecular parameters as independent variables, and age and BMI as covariates. Age and BMI were selected as core covariates because of their established relationships with bone density. For families of multiple comparisons, the Benjamini–Hochberg procedure was used to control the false discovery rate (FDR). FDR correction was applied separately to the 10 overall between-group osteoimmune biomarker comparisons, the 30 between-group comparisons across DXA-defined bone-status subgroups, and the 45 exploratory univariable biomarker–bone associations (nine biomarkers across five imaging-derived bone-density outcomes). Missing data were not imputed. Analyses were performed using available-case data; accordingly, the number of participants included in individual analyses varied according to data availability. For multivariable analyses, complete cases for all variables included in the respective model were used. All tests were two-sided and statistical significance was defined as *p* < 0.05 for unadjusted analyses and q < 0.05 for FDR-adjusted analyses.

## Results

### Study population and clinical characteristics

Age, BMI, and the distribution of DXA-defined bone-status categories were comparable between SSc and non-SSc groups (Table 1). Premenopausal status was present in 9 SSc patients and 12 non-SSc controls. Mean age at menopause was lower in SSc group, however, the difference did not reach statistical significance. Across the entire cohort osteoporosis was present in 23, osteopenia in 37, and normal bone status in 25 participants.

Vitamin D supplementation was more frequent in SSc patients than in controls (40/40 [100.0%] vs. 25/45 [55.6%], *p* < 0.001), whereas antiosteoporotic treatment was similarly distributed between groups (17.5% vs. 20.0%, respectively) (Table 1).

Within the SSc group, numbers of lcSSc and dcSSc patients were almost equal. Mean disease duration was 3.88 ± 2.2 years, and was comparable across DXA-defined bone status subgroups.

Gastrointestinal involvement assessed by the UCLA SCTC GIT 2.0 questionnaire was generally mild in SSc patients, with the highest mean GIT score observed in osteopenic patients (median: 0.39 [range: 0–1.51]), followed by those with normal bone status (0.26, [0–0.66]) and osteoporosis (0.19 [0–0.47]). Non-SSc controls showed lower overall GIT scores (0.18 [0–1.05]), with minimal variation across bone status categories.

DXA-derived parameters did not differ significantly between groups, consistent with the broadly comparable distribution of DXA-defined bone-status categories. FRAX-derived fracture risk estimates, calculated with FN BMD, were numerically higher in SSc patients, although between-group differences were not statistically significant.

**Table 1:** Baseline demographic, clinical and bone related characteristics of SSc patients and non-SSc controls

Parameters				SSc (*n* = 40)		Non-SSc (*n* = 45)		*p* value
Age (years), mean ± SD				57.2 ± 9.64		59.9 ± 11.49		0.266
BMI (kg/m^2^), mean ± SD				26.1 ± 4.56		27.3 ± 4.71		0.259
Bone status groups		Osteoporosis (OP), n (%)		12 (30.0)		11 (24.4)		0.629
		Osteopenia (OPe), n (%)		18 (45.0)		19 (42.2)		0.829
		Normal bone, n (%)		10 (25.0)		15 (33.3)		0.478
SSc duration, months, mean ± SD				45.2 ± 24.56		–		–
Age at SSc diagnosis, mean ± SD				53.4 ± 9.92		–		–
SSc subtype		lcSSc, n (%)		21 (52.5)		–		–
		dcSSc, n (%)		19 (47.5)		–		–
Patients in menopause, n (%)				31 (77.5)		34 (75.6)		0.999
Menopausal age (years), mean ± SD				46.69 ± 5.62		49.35 ± 4.68		0.115
Duration of menopause (years), mean ± SD				14.10 ± 8.44		16.03 ± 8.76		0.381
Current smoking, n (%)				3 (7.5)		6 (13.3)		0.494
Alcohol intake (occasionally), n (%)				13 (32.5)		17 (37.8)		0.655
Treatments n (%)		Corticosteroid (> 5 mg methylprednisolone)		0 (0)		0 (0)		–
		MMF (current)		25 (62.5)		–		–
		Tocilizumab (current)		6 (15)		–		–
		Rituximab (current)		4 (10)		–		–
		Cyclophosphamide (former)		11 (27.5)		–		–
		Bisphosphonates (current)		4 (10.0)		5 (11.1)		0.868
		Denosumab (current)		3 (7.5)		4 (8.9)		0.816
		D-vitamin supplementation		40 (100.0)		25 (55.6)		**< 0.0001**
		Calcium supplementation		12 (30.0)		15 (33.3)		0.818
UCLA GIT 2.0 total score, median (Q1-Q3)				0.22 (0.06–0.45)		0.11 (0.03–0.20)		0.087
FRAX major osteoporotic %, median (Q1-Q3)				6.3 (3.75–10.25)		4.9 (2.70–8.73)		0.237
FRAX hip %, median (Q1-Q3)				1.15 (0.40–3.70)		0.7 (0.30–1.95)		0.186
DXA BMD	L1–4 (g/cm^2^), mean ± SD		OP, mean	1.128 ± 0.205	0.849	1.141 ± 0.192	0.952	0.369
			OPe, mean		1.100		1.124	
			normal, mean		1.295		1.370	
	FN (g/cm^2^), mean ± SD		OP, mean	0.841 ± 0.153	0.699	0.852 ± 0.127	0.727	0.578
			OPe, mean		0.825		0.822	
			normal, mean		1.020		0.983	
DXA T-score	L1–4, mean ± SD		OP, mean	− 0.50 ± 1.65	− 2.35	− 0.40 ± 1.51	− 1.88	0.877
			OPe, mean		− 0.71		− 0.47	
			normal, mean		0.91		1.55	
	FN, mean ± SD		OP, mean	− 1.22 ± 1.28	− 2.40	− 1.05 ± 1.07	− 2.12	0.472
			OPe, mean		− 1.30		− 1.31	
			normal, mean		0.34		0.06	

Distal radius pQCT			SSc (*n* = 31)		non-SSc (*n* = 35)		*p* value	
pQCT vBMD	Total (mg/cm^3^); mean ± SD	OP, mean	293.37 ± 75.80	235.7	311.76 ± 55.51	260.7	0.201	0.128
		OPe, mean		285.3		311.2		0.126
		normal, mean		366.4		353.6		0.321
	Trabecular (mg/cm^3^); mean ± SD	OP, mean	150.83 ± 42.86	113.7	178.29 ± 43.35	156.6	**0.014**	**0.023**
		OPe, mean		151.0		169.7		0.095
		normal, mean		187.4		209.4		0.228
	Cortical+subcortical (mg/cm^3^); mean ± SD	OP, mean	409.65 ± 117.38	335.1	420.64 ± 80.59	345.6	0.603	0.305
		OPe, mean		394.9		426.5		0.125
		normal, mean		512.1		471.2		0.424
pQCT T-score	Total, mean ± SD	OP, mean	− 1.55 ± 1.27	− 2.5	− 1.13 ± 1.06	− 2.1	0.144	0.423
		OPe, mean		− 1.68		− 1.03		0.054
		normal, mean		− 0.33		− 0.52		0.188
	Trabecular, mean ± SD	OP, mean	− 1.27 ± 1.07	− 2.2	− 0.59 ± 1.09	− 1.13	**0.011**	**0.013**
		OPe, mean		− 1.27		− 0.81		0.093
		normal, mean		− 0.36		0.19		0.260
pQCT Z-score	Total, mean ± SD	OP, mean	− 0.72 ± 1.12	− 1.40	− 0.19 ± 0.86	− 0.74	**0.031**	**0.020**
		OPe, mean		− 0.76		− 0.07		0.231
		normal, mean		0.03		0.04		0.986
	Trabecular, mean ± SD	OP, mean	− 0.87 ± 0.96	− 1.60	− 0.09 ± 0.99	− 0.46	**0.002**	**0.005**
		OPe, mean		− 0.82		− 0.23		0.051
		normal, mean		− 0.21		0.43		0.183

Osteoimmune markers	SSc (*n* = 38)	non-SSc (*n* = 45)	*p* value	q-value
OPG (pg/ml), mean ± SD	2248.31 ± 899.48	1897.34 ± 539.90	**0.0001**	**0.0003**
OPN (pg/ml), mean ± SD	7126.58 ± 861.40	5862.92 ± 465.89	0.346	0.384
PDGF-BB (pg/ml), mean ± SD	23286.45 ± 4470.38	14845.11 ± 8470.60	**0.035**	**0.0438**
ACP5 (pg/ml), mean ± SD	7441.98 ± 466.23	5775.81 ± 2086.75	**0.0003**	**0.0006**
RANKL (pg/ml), mean ± SD	367.91 ± 179.18	160.89 ± 119.67	**< 0.0001**	**< 0.0003**
TNF-α (pg/ml), mean ± SD	33.22 ± 29.91	27.54 ± 15.68	0.627	0.627
IL-6 (pg/ml), mean ± SD	105.55 ± 98.31	43.49 ± 21.83	**< 0.0001**	**< 0.0003**
IL-1β (pg/ml), mean ± SD	16.63 ± 16.06	8.07 ± 8.71	**0.023**	**0.0383**
DKK-1 (pg/ml), mean ± SD	1000.01 ± 356.40	618.62 ± 368.58	**< 0.0001**	**< 0.0003**
RANKL/OPG, mean ± SD	0.19 ± 0.26	0.11 ± 0.07	**0.031**	**0.0438**

SSc: systemic sclerosis; lcSSc: limited cutaneous systemic sclerosis; dcSSc: diffuse cutaneous systemic sclerosis; BMI: body mass index; OP: osteoporosis; OPe: osteopenia; FRAX: Fracture Risk Assessment Tool; UCLA GIT 2.0: University of California Los Angeles Scleroderma Clinical Trial Consortium Gastrointestinal Tract Instrument 2.0; MMF: mycophenolate mofetil; ACP5: acid phosphatase 5 (tartrate-resistant acid phosphatase 5b); DKK-1: Dickkopf-related protein 1; IL: interleukin; OPG: osteoprotegerin; OPN: osteopontin; PDGF-BB: platelet-derived growth factor-BB; RANKL: receptor activator of nuclear factor κB ligand; TNF-α: tumor necrosis factor alpha; DXA: dual-energy X-ray absorptiometry; pQCT: peripheral quantitative computed tomography; BMD: bone mineral density; FN: femoral neck; SD: standard deviation

Values are presented as mean ± standard deviation (SD), median (interquartile range, Q1–Q3), or number (percentage), as appropriate. Bone status categories (osteoporosis, osteopenia, and normal bone status) were defined according to DXA-based WHO T-score criteria. *P* values were calculated using independent-samples t test or Mann–Whitney U test for continuous variables and χ^2^ or Fisher’s exact test for categorical variables, as appropriate. *P* values refer to two-sided comparisons between SSc patients and non-SSc controls. For the osteoimmune biomarker panel, Benjamini–Hochberg FDR correction was applied across the 10 between-group comparisons. Statistical significance was defined as *p* < 0.05 for unadjusted analyses and q < 0.05 for FDR-adjusted analyses. Bold values indicate statistically significant results (*p* < 0.05 for nominal comparisons and q < 0.05 for FDR-adjusted comparisons, as applicable)

### pQCT-derived vBMD and correlation with DXA parameters

**Fig. 1:**
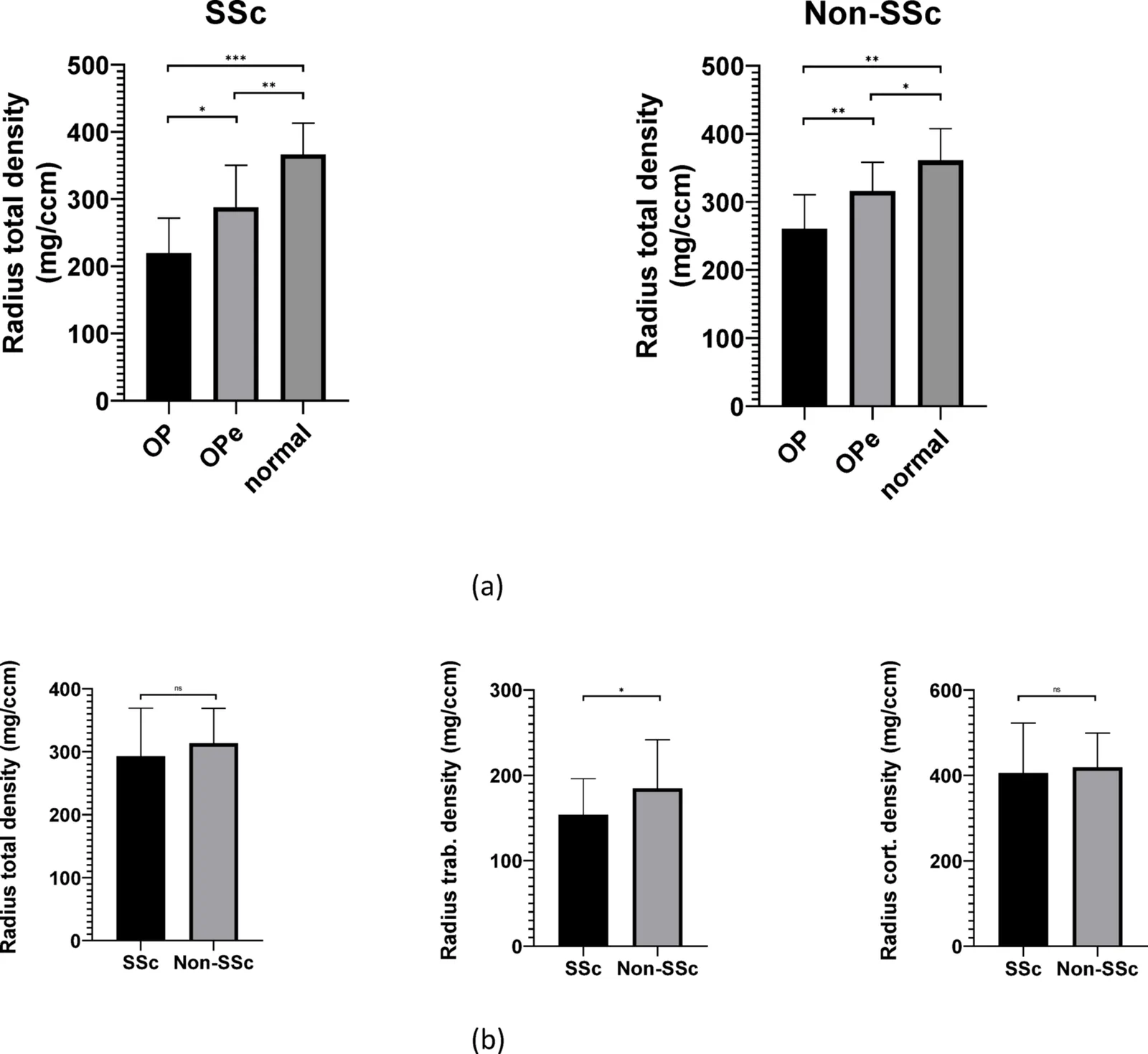
**a** Distal radius pQCT-derived volumetric bone density across DXA-defined bone status categories in SSc patients (left panel) and non-SSc controls (right panel). Bar graphs depict mean (± SD). Participants were stratified according to DXA-derived bone status into osteoporotic (OP), osteopenic (OPe), and normal bone status subgroups. *p < 0.05, **p < 0.01, ***p < 0.001. Graphs were created using GraphPad Prism 8.0.1. **b** Comparison of distal radius pQCT-derived volumetric bone density (vBMD) between SSc patients and non-SSc controls. Bar graphs show mean (± SD) total, trabecular and cortical + subcortical vBMD of the distal radius in SSc patients and non-SSc control subjects. *p < 0.05. Graphs were created using GraphPad Prism 8.0.1

Distal radius pQCT measurements were available for 31 SSc patients and 35 controls. Missing pQCT data were evenly distributed across bone status subgroups. Among SSc patients, those with and without available pQCT measurements did not differ significantly in age, BMI, disease duration, SSc subtype, menopausal status, or DXA-defined bone status. Given normal data distribution, Pearson correlation was applied. In both cohorts, pQCT parameters showed a clear stepwise gradient across DXA-defined bone status categories (Table 1; Fig. 1a).

Total and cortical+subcortical vBMD did not differ significantly between SSc patients and controls, while trabecular vBMD was significantly lower in SSc patients, both as absolute density and as T- and Z-scores (Table 1; Fig. 1b).

Prevalent MOF was identified in 12 of 40 (30.0%) SSc patients and 8 of 45 (17.8%) controls, with no statistically significant difference between the groups (*p* = 0.209). Among the 31 SSc patients with available pQCT data, 11 had a history of prevalent MOF, whereas 20 had no prevalent MOF. Patients with prevalent MOF had significantly lower total vBMD (237.5 ± 54.6 vs. 314.7 ± 73.9 mg/cm^3^, *p* = 0.005) and trabecular vBMD (112.2 ± 36.0 vs. 168.6 ± 36.5 mg/cm^3^, *p* = 0.0003) than those without MOF. Cortical+subcortical vBMD was also significantly lower in patients with prevalent MOF (median 340.1 vs. 437.0 mg/cm^3^, *p* = 0.020).

**Fig. 2:**
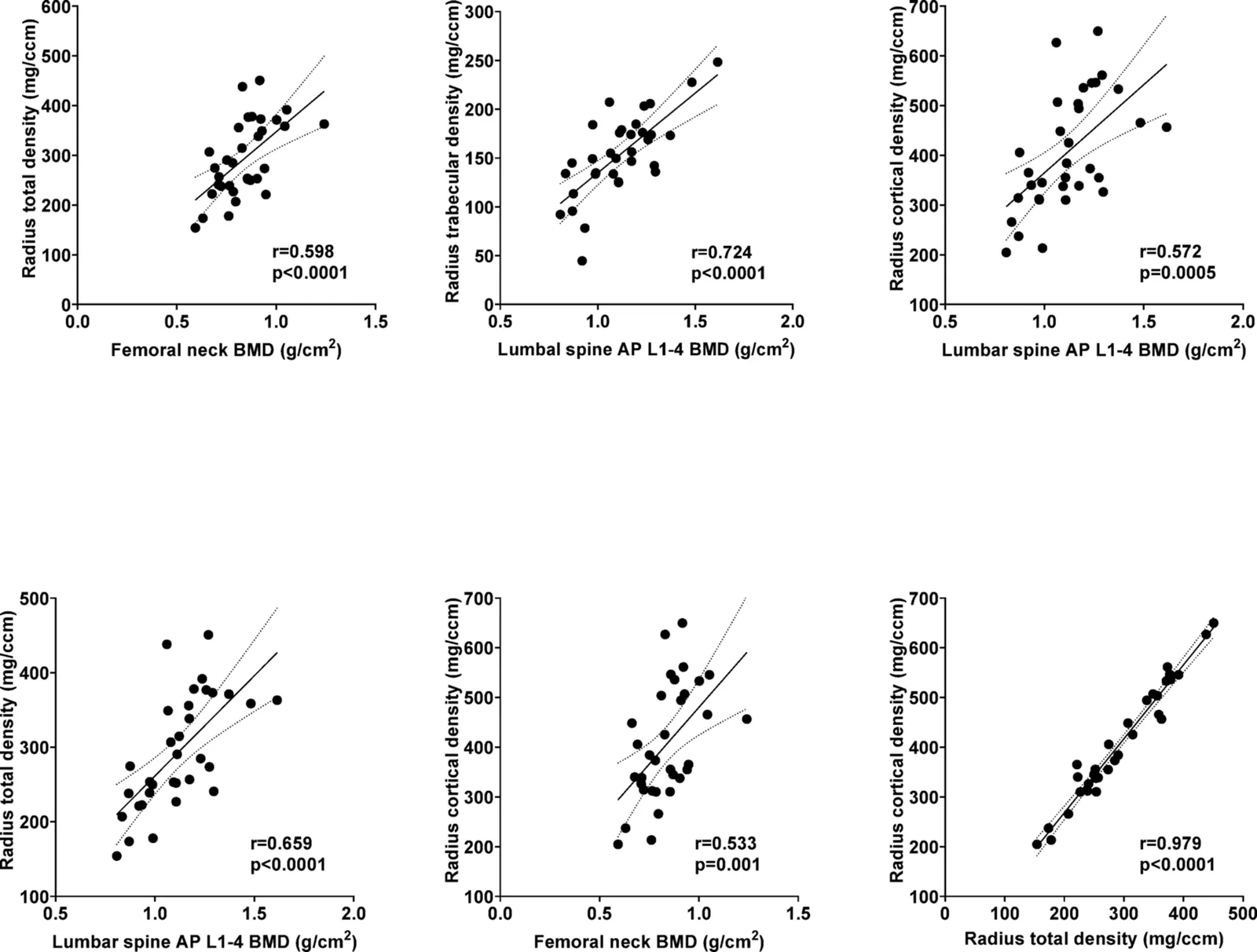
Correlations between DXA-derived areal BMD and pQCT-derived volumetric bone density parameters. Scatter plots illustrate correlations between lumbar spine (L1-L4) and femoral neck areal bone mineral density (aBMD) measured by dual-energy X-ray absorptiometry (DXA) and distal radius volumetric bone mineral density (vBMD) parameters assessed by peripheral quantitative computed tomography (pQCT). Pearson correlation coefficients (r) and corresponding *p* values are shown in each panel; dashed lines indicate linear regression with 95% confidence intervals. Graphs were created using GraphPad Prism 8.0.1

In patients with SSc, L1-4 BMD showed strong positive correlations with trabecular pQCT density, and also correlated with total and cortical+subcortical volumetric density at the radius. FN BMD correlated with both total and cortical+subcortical pQCT density. In addition, total volumetric density was very strongly correlated with cortical+subcortical density, indicating a close coupling between these pQCT-derived compartments. All correlations were statistically significant (Fig. 2).

### Routine laboratory markers of bone and mineral metabolism

Routine laboratory markers largely fell within reference ranges in both groups, with a low and comparable frequency of values outside normal limits. Serum osteocalcin was modestly lower in SSc patients than in controls (16.23 ± 7.67 vs. 19.50 ± 7.26 µg/L, *p* = 0.029), although all values remained within the reference range. Serum 25-hydroxyvitamin D levels were higher in SSc patients (92.65 ± 28.80 vs. 83.16 ± 28.79 nmol/L, *p* = 0.031), reflecting more frequent supplementation. Serum 25-hydroxyvitamin D concentrations below 75 nmol/L were observed in 10/40 (25.0%) SSc patients and 18/45 (40.0%) controls. None of the observed differences in routine laboratory markers remained significant after Benjamini-Hochberg FDR correction (Online Resource SI3).

### Circulating bone-related molecular markers

SSc patients exhibited a distinct circulating osteoimmune profile, characterized by higher levels of OPG, RANKL, ACP5, and PDGF-BB, increased Wnt-pathway inhibition marker DKK-1, and elevated inflammatory cytokines IL-6 and IL-1β compared with controls. The RANKL/OPG ratio was also higher in SSc (*p* < 0.05), whereas TNF-α and OPN did not differ between groups (Table 1). After Benjamini–Hochberg FDR correction across the targeted osteoimmune biomarker panel, the between-group differences in OPG, PDGF-BB, ACP5, RANKL, IL-6, IL-1β, DKK-1, and the RANKL/OPG ratio remained statistically significant. Two samples were excluded due to gross lipemia. Because tocilizumab treatment may influence circulating IL-6 concentrations, a sensitivity analysis was performed excluding the six SSc patients receiving tocilizumab. After their exclusion, circulating IL-6 levels remained significantly higher in patients with SSc than in controls (*n* = 32 vs. *n* = 45; median 67.22 [IQR 57.82–97.16] vs. 42.50 [28.90–53.13] pg/mL; Mann–Whitney U = 1123.0, *p* < 0.0001).

Subgroup analyses across osteoporosis, osteopenia, and normal bone status showed consistent elevation of DKK-1 levels in SSc patients, with additional subgroup-specific differences in OPG, ACP5, RANKL, IL-6, and IL-1β levels. In exploratory analyses stratified by DXA-defined bone status, FDR-adjusted between-group differences were observed for ACP5, RANKL, and DKK-1 in the osteopenia subgroup and for IL-1β in the osteoporosis subgroup, with higher levels in SSc patients than in the corresponding non-SSc groups (Table 2).

**Table 2:** Osteoimmune biomarker profiles in SSc and non-SSc participants stratified by DXA-defined bone status

Marker (pg/ml)	SSc, OP, mean ± SD *n* = 12	Non-SSc, OP, mean ± SD *n* = 11	*p* value	q-value	SSc, OPe, mean ± SD *n* = 17	Non-SSc, OPe, mean ± SD *n* = 19	*p* value	q-value	SSc, *N*, mean ± SD *n* = 9	Non-SSc, *N*, mean ± SD *n* = 15	*p* value	q-value
OPG	2240.54 ± 708.35	1710.07 ± 695.37	**0.032**	0.113	2485.40 ± 1081.34	2320.47 ± 2227.00	**0.026**	0.111	1830.90 ± 614.82	1498.70 ± 386.45	0.091	0.182
OPN	7435.71 ± 515.85	6427.41 ± 249.41	0.730	0.782	6926.43 ± 4621.79	6845.18 ± 3777.24	0.659	0.760	7115.89 ± 5577.18	4204.76 ± 1101.41	0.129	0.242
PDGF-BB	18591.75 ± 14451.16	7005.53 ± 808.51	0.169	0.277	24993.80 ± 14177.48	20081.36 ± 20478.38	0.425	0.531	25846.86 ± 15164.14	13961.55 ± 9441.94	0.176	0.277
ACP5	7616.83 ± 2915.90	5546.37 ± 1609.06	**0.026**	0.111	8023.11 ± 2100.88	6146.31 ± 2438.36	**0.007**	**0.049**	6186.12 ± 2271.36	5414.78 ± 2281.72	0.311	0.467
RANKL	234.35 ± 116.45	150.27 ± 97.87	0.059	0.136	539.35 ± 97.93	212.79 ± 181.52	**0.003**	**0.038**	237.33 ± 101.93	199.58 ± 170.99	0.064	0.136
TNF-α	43.84 ± 39.45	31.36 ± 14.56	0.898	0.898	26.46 ± 26.32	24.32 ± 17.36	0.726	0.782	32.99 ± 20.97	28.82 ± 14.37	0.851	0.880
IL-6	153.11 ± 151.28	35.29 ± 19.42	**0.034**	0.113	93.90 ± 59.55	43.63 ± 20.78	**0.049**	0.133	69.97 ± 42.29	49.33 ± 24.16	0.138	0.244
IL-1β	19.49 ± 14.98	5.53 ± 5.01	**0.006**	**0.049**	17.91 ± 8.99	7.22 ± 4.04	0.360	0.514	10.72 ± 10.27	11.41 ± 13.98	0.504	0.605
DKK-1	980.97 ± 451.06	550.70 ± 296.02	**0.013**	0.075	1010.64 ± 333.08	650.94 ± 401.58	**0.003**	**0.038**	1003.70 ± 302.45	627.48 ± 389.75	**0.048**	0.133
RANKL/OPG	0.11 ± 0.06	0.10 ± 0.07	0.379	0.517	0.32 ± 0.03	0.10 ± 0.07	0.056	0.136	0.14 ± 0.09	0.12 ± 0.08	0.411	0.531

ACP5: acid phosphatase 5 (tartrate-resistant acid phosphatase 5b); DKK-1: Dickkopf-related protein 1; IL: interleukin; OPG: osteoprotegerin; OPN: osteopontin; PDGF-BB: platelet-derived growth factor-BB; RANKL: receptor activator of nuclear factor κB ligand; TNF-α: tumor necrosis factor alpha. Data are presented as mean ± standard deviation (SD)

Values are presented as mean ± SD. *P* values represent nominal between-group comparisons between SSc and non-SSc participants within each DXA-defined bone-status category. To account for multiple testing, the Benjamini–Hochberg false discovery rate correction was applied across the 30 subgroup comparisons presented in the table. q < 0.05 was considered statistically significant. Bold values indicate statistically significant results (*p* < 0.05 for nominal comparisons and q < 0.05 for FDR-adjusted comparisons, as applicable). Subgroup analyses were exploratory

### Associations with clinical characteristics

Within the SSc cohort, OPG levels correlated positively with age (*r* = 0.363, *p* = 0.022) (Online Resource SI5), age at diagnosis (*r* = 0.393, *p* = 0.012), FRAX major osteoporotic fracture probability (*r* = 0.328, *p* = 0.015), and FRAX hip fracture probability (*r* = 0.364, *p* = 0.021). The associations with FRAX probabilities were considered exploratory. No significant associations were detected between the exploratory serum molecules and BMI, disease duration, or gastrointestinal involvement.

### Associations with bone parameters

In univariate linear regression analyses, higher circulating OPG levels were inversely associated with L1-4 aBMD and FN aBMD. Higher IL-6 levels also showed significant inverse associations with FN and L1-4 aBMD, as well as with radius total and trabecular vBMD, while ACP5 showed a significant negative relationship with L1-4 aBMD and radius trabecular vBMD (Table 3).

After adjustment for age and BMI, the associations between OPG and DXA-derived aBMD parameters were attenuated and no longer statistically significant (*p* = 0.27 and *p* = 0.39, respectively), whereas age remained associated with lower BMD at both skeletal sites, suggesting that the relationship between OPG and bone integrity is largely age-dependent.

In exploratory multivariable models adjusted for age and BMI, IL-6 remained inversely associated with radius trabecular vBMD. The association between IL-6 and FN aBMD, L1-4 aBMD, as well as radius total vBMD was attenuated after adjustment for age and BMI (*p* = 0.086, *p* = 0.109 and *p* = 0.066, respectively). ACP5 remained inversely associated with L1-4 aBMD and radius trabecular vBMD. In these models, age was also associated with bone density, while BMI was not a significant predictor.

Across the broader set of biomarker–bone comparisons, several nominal associations were observed; however, none remained statistically significant after Benjamini–Hochberg FDR correction. Accordingly, the biomarker–bone associations should be interpreted as exploratory.

**Table 3:** Uni- and multivariable associations between circulating biomarkers and imaging-derived BMD parameters in patients with systemic sclerosis (*n* = 38)

Dependent variable	Independent variable	Univariable linear regression			Multivariable linear regression		
		B (95% CI)	β	*p* value	B (95% CI)	β	*p* value
DXA L1-4 aBMD	OPG	− 0.092 (− 0.180 to − 0.004)	− 0.34	0.041	–	–	–
	IL-6	− 0.824 (− 1.457 to − 0.191)	− 0.41	0.013	–	–	–
	ACP5	− 0.029 (− 0.056 to − 0.002)	− 0.35	0.039	− 0.024 (− 0.052 to − 0.003)	− 0.31	0.045
	Age	− 0.011 (− 0.018 to − 0.004)	− 0.49	0.003	− 0.0085 (− 0.0155 to − 0.00160)	− 0.40	0.018
DXA Fn aBMD	OPG	− 0.071 (− 0.139 to − 0.003)	− 0.31	0.041	–	–	–
	IL-6	− 0.591 (− 1.089 to − 0.094)	− 0.38	0.021	–	–	–
	Age	− 0.011 (− 0.015 to − 0.006)	− 0.63	< 0.001	–	–	–
Radius tot. vBMD	IL-6	− 0.258 (− 0.499 to − 0.017)	− 0.37	0.037	–	–	–
	Age	− 3.174 (− 5.689 to − 0.658)	− 0.41	0.015	–	–	–
Radius trab. vBMD	IL-6	− 0.173 (− 0.299 to − 0.047)	− 0.35	0.008	− 0.146 (− 0.277 to − 0.014)	− 0.16	0.031
	Age	− 1.948 (− 3.205 to − 0.690)	− 0.48	0.003	− 1.540 (− 2.859 to − 0.221)	− 0.34	0.024
	ACP5	− 7.033 (− 12.156 to − 1.910)	− 0.44	0.016	− 5.687 (− 11.032 to − 0.342)	− 0.35	0.038
	Age	− 1.948 (− 3.205 to − 0.690)	− 0.48	0.003	− 1.353 (− 2.697 to − 0.009)	− 0.35	0.049

aBMD: areal bone mineral density; ACP5: acid phosphatase 5 (tartrate-resistant acid phosphatase: TRAP-5b); IL-6: interleukin-6; OPG: osteoprotegerin; DXA: dual-energy X-ray absorptiometry; FN: femoral neck; pQCT: peripheral quantitative computed tomography; tot. vBMD: total volumetric bone mineral density; trab. vBMD: trabecular volumetric bone mineral density, B: unstandardized regression coefficient; β: standardized regression coefficient; CI: confidence interval

Uni- and multivariable linear regression analyses were performed to assess associations between circulating biomarkers and bone density parameters. B represents the unstandardized regression coefficient with 95% confidence intervals (CI), while β denotes the standardized regression coefficient. Negative B values indicate inverse associations between the independent variable and bone density outcomes. Multivariable models were adjusted for age and BMI and were considered exploratory. Nominal *p* values are presented for the individual models. Across the broader set of 45 univariable biomarker–bone comparisons, Benjamini–Hochberg FDR correction was applied; none remained statistically significant at q < 0.05

## Discussion

In this study, we investigated skeletal involvement in women with relatively early SSc by integrating conventional DXA and FRAX assessment with compartment-specific pQCT and osteoimmunological profiling. Three principal findings emerged: preferentially lower trabecular radial vBMD in SSc despite broadly comparable DXA-defined bone status; a distinct circulating osteoimmune profile with multiple between-group differences remaining significant after FDR correction; and significantly lower radial vBMD across all measured compartments in SSc patients with prevalent major osteoporotic fractures. Exploratory analyses further identified associations of IL-6 and ACP5 with trabecular-rich bone parameters.

The composition of the control cohort should be considered when interpreting the imaging findings. Controls were frequency-matched to the SSc cohort by age, while representation across DXA-defined bone-status categories was balanced during recruitment to allow comparisons between SSc and non-SSc participants with broadly comparable conventional skeletal status. Consequently, the absence of significant between-group differences in DXA-derived BMD should not be interpreted as evidence of preserved bone mass in SSc or as demonstrating greater sensitivity of pQCT over DXA. Rather, this design allowed us to explore whether compartment-specific skeletal differences were detectable in SSc in the context of broadly comparable DXA-defined bone status. Within this framework, the significantly lower trabecular vBMD observed in SSc is of particular interest.

Distal radius pQCT complemented DXA-derived BMD by providing volumetric and compartment-specific information. Moderate to strong correlations between pQCT-derived volumetric density and central DXA measures support convergent validity, while the lower trabecular vBMD and more negative trabecular Z-scores observed in SSc patients indicate preferential involvement of the trabecular compartment, consistent with prior reports. The preferential involvement of trabecular bone is biologically plausible given its higher metabolic activity and sensitivity to inflammatory, vascular, and metabolic influences that characterize SSc. Importantly, Z-scores were calculated using a Caucasian reference population provided by the manufacturer, supporting the validity of age-adjusted comparisons in this cohort [10]. These findings indicate that pQCT provides compartment-specific skeletal information complementary to standard DXA measures, particularly in patients with relatively short disease duration, and suggest that SSc patients may exhibit subtle deviations from age-expected peripheral bone density [28].

Importantly, the availability of prevalent fracture data allowed us to relate compartment-specific pQCT abnormalities to a clinically relevant skeletal outcome. SSc patients with a history of major osteoporotic fracture showed significantly lower total, trabecular, and cortical+subcortical radial vBMD compared with patients without prevalent MOF, with a particularly marked difference observed in the trabecular compartment. These findings provide complementary information on skeletal fragility alongside conventional DXA and FRAX-based assessment. However, given the limited number of fracture events and the cross-sectional nature of the study, these findings should be considered exploratory and require confirmation in larger prospective cohorts.

FRAX estimates were numerically higher in SSc, although the between-group differences were not statistically significant. Epidemiologic studies nevertheless indicate an increased fracture risk in SSc [4]. DXA-derived BMD remains central to fracture risk assessment and is incorporated into FRAX [6]. However, FRAX does not capture SSc-specific biology (e.g. systemic inflammation, microvascular dysfunction, malabsorption, reduced mechanical loading), nor does it incorporate compartmental bone information [8–10]. The integration of DXA, FRAX, and pQCT highlights complementary aspects of skeletal health [7, 8]. While DXA and FRAX appropriately identify fracture risk, pQCT may add relevant structural detail. This multimodal approach may support a more nuanced understanding of bone involvement in SSc, by integrating fracture risk prediction with detailed structural characterization.

Routine markers of mineral metabolism and bone turnover largely remained within reference ranges and showed substantial overlap between groups, and differences did not persist after FDR correction, suggesting that standard biochemical parameters may be insensitive to disease-specific skeletal alterations in systemic inflammatory conditions. Inflammatory, vascular, and signaling abnormalities may contribute to SSc-related bone involvement without overt disturbances in systemic mineral homeostasis, which may partly explain the limited discriminatory value of routine serum markers in this setting [29].

In contrast, the targeted molecular panel revealed robust differences: SSc patients showed higher OPG, RANKL, ACP5, IL-6, IL-1β and DKK-1 levels, with a modest but significant increase in the RANKL/OPG ratio. This constellation is consistent with dysregulated immune–bone crosstalk rather than isolated disturbances in mineral homeostasis. Collectively, these findings suggest that SSc is associated with a distinct circulating bone-related and immunoregulatory biomarker profile [30, 31].

The consistent elevation of OPG together with increased RANKL and ACP5 levels points to an activated but dysregulated osteoclast-related signaling milieu, rather than a simple shift toward bone resorption. This pattern is in line with the concept of uncoupled bone remodeling, previously suggested in chronic inflammatory and autoimmune conditions [32]. The RANKL-OPG system represents a central regulatory axis of osteoclastogenesis and bone resorption. Elevated OPG levels in SSc may reflect a compensatory response to increased osteoclastogenic drive or inflammatory signaling, rather than effective inhibition of bone resorption [33].

Elevated circulating IL-6 and IL-1β levels in SSc are compatible with an inflammatory milieu potentially relevant to bone remodeling. Both cytokines are known to promote osteoclast differentiation and activity [4, 32]. Notably, TNF-α levels did not differ between groups, indicating heterogeneity in the circulating inflammatory profile. Circulating IL-6 concentrations should be interpreted cautiously because six patients were receiving tocilizumab, which may increase circulating IL-6 through reduced receptor-mediated clearance. Importantly, however, IL-6 levels remained significantly higher in SSc after exclusion of all tocilizumab-treated patients (*p* < 0.0001), indicating that the observed between-group difference was not attributable solely to tocilizumab exposure. Nevertheless, circulating IL-6 should not be interpreted as a direct measure of endogenous IL-6 pathway activity or skeletal inflammation.

Our findings also highlight a marked elevation of DKK-1 in SSc patients. DKK-1 is a potent inhibitor of canonical Wnt signaling, a pathway essential for osteoblast differentiation and bone formation. Increased DKK-1 expression has been implicated in uncoupling bone formation from resorption in inflammatory diseases, thereby promoting net bone loss [20, 32, 34]. In the bone-status subgroup analyses, DKK-1 levels were nominally higher in SSc patients across the examined categories; however, after FDR correction, the difference remained significant only in the osteopenic subgroup. These findings should therefore be interpreted cautiously, but are compatible with altered Wnt-mediated regulation of bone formation in SSc. Given the reported associations of higher circulating DKK-1 levels with fibrotic manifestations (skin and lung) [35, 36] and the established anti-osteogenic effects of DKK-1 [37], these observations raise the hypothesis that DKK-1 may be relevant to both fibrotic disease manifestations and skeletal alterations in SSc.

Within SSc, OPG correlated with chronological age and age at diagnosis, consistent with the known age-dependence of OPG biology [38]. Higher circulating OPG levels were associated with lower L1-4 and FN aBMD in univariable analyses; however, these associations were no longer significant after adjustment for age, suggesting substantial age dependence. The observed correlations between OPG and FRAX probabilities should also be interpreted cautiously. Because FN aBMD was incorporated into FRAX calculations and OPG was associated with FN aBMD in our cohort, these correlations may partly reflect the mathematical structure of FRAX rather than an independent relationship between OPG and fracture probability. Beyond bone metabolism, OPG has been implicated in vascular homeostasis and may increase as a compensatory response to endothelial dysfunction, vascular injury, and inflammatory signaling. Given the central role of microvascular pathology in SSc, elevated circulating OPG may therefore reflect an interface between skeletal, inflammatory, and vascular processes rather than osteoclast-related signaling alone [33, 35]. Thus, the increased OPG levels observed here may represent a compensatory response to altered bone remodeling and/or vascular-inflammatory injury rather than a direct marker of osteoclastogenic activity.

In exploratory multivariable analyses, the inverse association between IL-6 and trabecular vBMD persisted after adjustment for age and BMI. Although this association did not remain significant after FDR correction across the broader biomarker–bone analysis and should therefore be interpreted cautiously, its localization to the trabecular compartment is biologically plausible given the established role of IL-6 in inflammatory bone remodeling.

Similarly, ACP5 showed inverse associations with L1-4 aBMD and radius trabecular vBMD, which persisted in exploratory models adjusted for age and BMI. Although these associations did not survive FDR correction and require confirmation, their preferential involvement of trabecular-rich skeletal sites is compatible with the established role of ACP5/TRAP-5b as a marker of osteoclast number and activity [39]. Given the higher metabolic activity of cancellous bone, this pattern provides a biologically plausible basis for further investigation of ACP5 in SSc-related skeletal alterations.

Subgroup analyses provided additional insight into disease heterogeneity. In patients with dcSSc, IL-6 showed significantly elevated serum levels compared to lcSSc patients (137.96 ± 126.95 vs. 76.38 ± 50.09 pg/ml, *p* = 0.003) (Online Resource SI4).

**Fig. 3:**
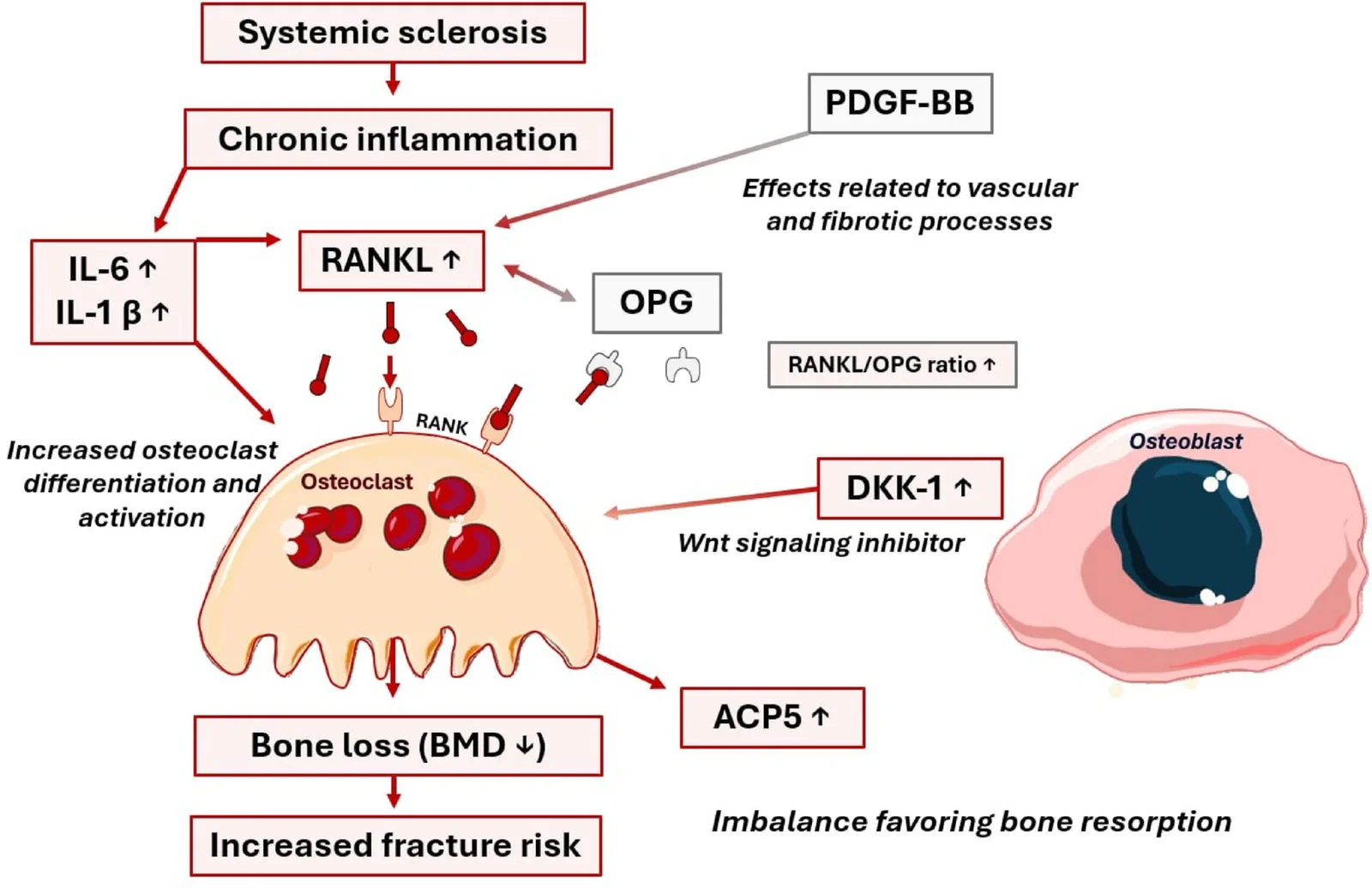
Conceptual overview of osteoimmunological pathways potentially associated with skeletal alterations in SSc. Abbreviations: IL: interleukin; RANKL: receptor activator of nuclear factor κB ligand; OPG: osteoprotegerin; PDGF-BB: platelet-derived growth factor-BB; DKK-1: Dickkopf-related protein 1; ACP5: acid phosphatase 5 (tartrate-resistant acid phosphatase 5b); BMD: bone mineral density

Our findings complement previous reports linking inflammatory and osteoclast-related biomarkers to skeletal involvement in SSc beyond traditional risk factors and support the concept that trabecular bone may represent a preferentially affected skeletal compartment in relatively early SSc. According to our results, the combined use of DXA and pQCT may allow a more nuanced assessment of compartment-specific bone alterations, while the observed associations of IL-6 and ACP5 with trabecular-rich bone parameters provide hypothesis-generating evidence for potential links between osteoimmune activity and skeletal alterations in SSc (Fig. 3).

Molecular alterations and reduced BMD have been consistently reported across inflammatory rheumatic diseases including rheumatoid arthritis, psoriatic arthritis, ankylosing spondylitis [40–42]. pQCT has been shown to provide compartment-specific assessment of trabecular and cortical bone beyond DXA-derived areal measures [43–45]. In contrast to these broader cohorts, our study focused on a clinically well-characterized, relatively early SSc population in order to explore skeletal alterations potentially present before the emergence of advanced disease-related structural and treatment-related confounders.

## Conclusion

In this cross-sectional study of women with relatively early SSc, we applied a multimodal approach integrating DXA, FRAX, pQCT, and an osteoimmunology-focused biomarker panel to characterize skeletal involvement. Three principal findings emerged: (I) pQCT revealed compartment-specific skeletal differences between SSc and non-SSc participants despite broadly comparable DXA-defined bone status, with preferential involvement of the trabecular compartment; (II) SSc was associated with a distinct circulating osteoimmune profile not captured by routine laboratory markers, and (III) SSc patients with prevalent major osteoporotic fractures had significantly lower radial vBMD across all measured compartments, supporting the clinical relevance of the observed pQCT abnormalities. Exploratory associations between selected osteoimmune biomarkers and DXA- and pQCT-derived BMD parameters were also observed, although these did not remain significant after correction for multiple testing and require confirmation in larger cohorts. Overall, our findings highlight preferential trabecular skeletal involvement in women with relatively early SSc and suggest that pQCT and osteoimmune profiling may provide complementary information alongside conventional DXA and clinical fracture-risk assessment.

## Limitations and future directions

Several limitations of this study should be acknowledged. First, the cross-sectional design does not allow evaluation of temporal relationships between SSc-related factors, changes in bone density, and fracture outcomes.

Second, although the study included well-characterized SSc patients and age-matched female controls, the sample size was modest, particularly in subgroup analyses. Furthermore, the clinic-based control cohort, with its deliberately balanced representation across DXA-defined bone-status categories, may not be representative of the general population. This sampling strategy also limits the ability of the present study to assess differences in DXA-derived BMD or osteoporosis prevalence between SSc patients and population-based controls. Two samples were excluded because of gross lipemia, further reducing the effective sample size. Although the two major SSc subtypes were in comparable proportions in the study cohort, the relatively small number of patients limited the interpretability of subtype-specific comparisons.

Third, pQCT measurements were limited to the distal radius, which may not fully reflect load-bearing regions such as the hip or spine.

Finally, vitamin D supplementation differed between groups, whereas antiosteoporotic treatment was similarly distributed. Although treatment reflects real-world clinical practice, its presence may have influenced BMD measurements and biomarker levels and may have attenuated observed group differences. Tocilizumab treatment may have influenced circulating IL-6 concentrations, although the between-group difference persisted after exclusion of tocilizumab-treated patients. Residual confounding by menopausal status, vitamin D supplementation, antiresorptive therapy, and immunomodulatory treatment cannot be excluded. Given the modest sample size, particularly for individual treatment exposures, adequately powered treatment-stratified or extensively adjusted multivariable analyses were not feasible.

Future studies with larger cohorts and treatment-stratified or longitudinal designs are needed to clarify the independent and additive value of pQCT and molecular markers in the assessment of skeletal involvement in SSc.

## Supplementary Information

Below is the link to the electronic supplementary material.

Supplementary Material 1

## References

[1] Lillpers K, McGuigan F, Andréasson K, Hesselstrand R, Kapetanovic MC, Åkesson KE (2025) Increased prevalence of osteopenia and osteoporosis in systemic sclerosis patients, including in men and young women-a case–control study. Rheumatology (Oxford) 64:2783–2791. 10.1093/rheumatology/keae543 PMC12048067 39388250

[2] Chen J, Lei L, Pan J, Zhao C (2020) A meta-analysis of fracture risk and bone mineral density in patients with systemic sclerosis. Clin Rheumatol 39:1181–1189. 10.1007/s10067-019-04847-0 31838641

[3] Marot M, Valéry A, Esteve E, Bens G, Müller A, Rist S, Toumi H, Lespessailles E (2015) Prevalence and predictive factors of osteoporosis in systemic sclerosis patients: a case-control study. Oncotarget 6:14865–14873. 10.18632/oncotarget.3806 PMC4558121 25944694

[4] Lai CC, Wang SH, Chen WS, Liu CJ, Chen TJ, Lee PC, Chang YS (2015) Increased risk of osteoporotic fractures in patients with systemic sclerosis: a nationwide population-based study. Ann Rheum Dis 74:1347–1352. 10.1136/annrheumdis-2013-204832 24532679

[5] Rogers B, Famenini S, Perin J, Danila MI, Wipfler K, Michaud K, McMahan ZH (2023) Clinical features associated with rate of fractures in patients with systemic sclerosis: a US cohort study. Arthritis Care Res (Hoboken) 75:2379–2388. 10.1002/acr.25137 PMC10836612 37093022

[6] Kanis JA, Hans D, Cooper C, Baim S, Bilezikian JP, Binkley N, Cauley JA et al (2011) Interpretation and use of FRAX in clinical practice. Osteoporos Int 22:2395–2411. 10.1007/s00198-011-1713-z 21779818

[7] Huang M, Hung VW, Li TK, Law SW, Wang Y, Chen S, Qin L (2021) Performance of HR-pQCT, DXA, and FRAX in the discrimination of asymptomatic vertebral fracture in postmenopausal Chinese women. Arch Osteoporos 16:125. 10.1007/s11657-021-00939-0 PMC8418592 34480663

[8] Lee KA, Kim HJ, Kim HS (2023) Comparison of predictive value of FRAX, trabecular bone score, and bone mineral density for vertebral fractures in systemic sclerosis: a cross-sectional study. Med (Baltim) 102:e32580. 10.1097/MD.0000000000032580 PMC9839281 36637920

[9] Kanis JA, Johansson H, McCloskey EV, Liu E, Schini M, Vandenput L, Åkesson KE et al (2025) Rheumatoid arthritis and subsequent fracture risk: an individual person meta-analysis to update FRAX. Osteoporos Int 36:653–671. 10.1007/s00198-025-07397-1 39955689

[10] Lochmüller EM, Müller R, Kuhn V, Lill CA, Eckstein F (2003) Can novel clinical densitometric techniques replace or improve DXA in predicting bone strength in osteoporosis at the hip and other skeletal sites? J Bone Min Res 18:906–912. 10.1359/jbmr.2003.18.5.906 12733731

[11] Mesinovic J, Breasail MÓ, Burt LA, Shore-Lorenti C, Zebaze R, Lim CQE, Ling Z et al (2024) Bone imaging modality precision and agreement between DXA, pQCT, and HR-pQCT. JBMR Plus 9:e158. 10.1093/jbmrpl/ziae158 PMC11752644 39845981

[12] Juhász B, Gulyás K, Horváth Á, Pethő Z, Bhattoa HP, Váncsa A, Szekanecz É et al (2017) Comparison of peripheral quantitative computed tomography forearm bone density versus DXA in rheumatoid arthritis patients and controls. Osteoporos Int 28:1271–1277. 10.1007/s00198-016-3850-x 27942777

[13] Horváth Á, Végh E, Pusztai A, Pethő Z, Hamar A, Czókolyová M, Bhattoa HP et al (2019) Complex assessment of bone mineral density, fracture risk, vitamin D status, and bone metabolism in Hungarian systemic sclerosis patients. Arthritis Res Ther 21:274. 10.1186/s13075-019-2072-y PMC6905018 31823821

[14] Hamar A, Hascsi Z, Pusztai A, Czókolyová M, Végh E, Pethő Z, Gulyás K et al (2021) Prospective, simultaneous assessment of joint and vascular inflammation by PET/CT in tofacitinib-treated patients with rheumatoid arthritis: associations with vascular and bone status. RMD Open 7:e001804. 10.1136/rmdopen-2021-001804 PMC8573670 34740980

[15] Omair MA, McDonald-Blumer H, Johnson SR (2014) Bone disease in systemic sclerosis: outcomes and associations. Clin Exp Rheumatol 32:S28–32 24321499

[16] Avouac J, Koumakis E, Toth E, Meunier M, Maury E, Kahan A, Cormier C et al (2012) Increased risk of osteoporosis and fracture in women with systemic sclerosis: a comparative study with rheumatoid arthritis. Arthritis Care Res (Hoboken) 64:1871–1878. 10.1002/acr.21761 22730393

[17] Filella X, Guañabens N (2023) Clinical use of bone markers: a challenge to variability. Adv Lab Med 5:7–14. 10.1515/almed-2023-0092 PMC11019881 38634081

[18] Vasikaran S, Eastell R, Bruyère O, Foldes AJ, Garnero P, Griesmacher A, McClung M et al (2011) Markers of bone turnover for the prediction of fracture risk and monitoring of osteoporosis treatment: a need for international reference standards. Osteoporos Int 22:391–420. 10.1007/s00198-010-1501-1 21184054

[19] Khosla S (2001) Minireview: the OPG/RANKL/RANK system. Endocrinology 142:5050–5055. 10.1210/endo.142.12.8536 11713196

[20] Steeve KT, Marc P, Sandrine T, Dominique H, Yannick F (2004) IL-6, RANKL, TNF-alpha/IL-1: interrelations in bone resorption pathophysiology. Cytokine Growth Factor Rev 15:49–60. 10.1016/j.cytogfr.2003.10.005 14746813

[21] Ruaro B, Casabella A, Paolino S, Pizzorni C, Ghio M, Seriolo C, Molfetta L et al (2018) Dickkopf-1 (Dkk-1) serum levels in systemic sclerosis and rheumatoid arthritis patients: correlation with the Trabecular Bone Score (TBS). Clin Rheumatol 37:3057–3062. 10.1007/s10067-018-4322-9 30291470

[22] Distler O, Highland KB, Gahlemann M, Azuma A, Fischer A, Mayes MD, Raghu G et al (2019) Nintedanib for systemic sclerosis–associated interstitial lung disease. N Engl J Med 380:2518–2528. 10.1056/NEJMoa1903076 31112379

[23] n Den Hoogen F, Khanna D, Fransen J, Johnson SR, Baron M, Tyndall A, Matucci-Cerinic M et al (2013) 2013 classification criteria for systemic sclerosis: an American college of rheumatology/European league against rheumatism collaborative initiative. Arthritis Rheum 65:2737–2747. 10.1002/art.38098 PMC3930146 24122180

[24] Volkmann ER, Andréasson K, Smith V (2024) Systemic sclerosis. Lancet 401:304–318. 10.1016/S0140-6736(22)01692-0 PMC9892343 36442487

[25] Khanna D, Hays RD, Maranian P, Seibold JR, Impens A, Mayes MD, Clements PJ et al (2009) Reliability and validity of the University of California, Los Angeles scleroderma clinical trial consortium gastrointestinal tract instrument. Arthritis Rheum 61:1257–1263. 10.1002/art.24730 PMC2767193 19714600

[26] Kanis JA, Adachi JD, Cooper C, Clark P, Cummings SR, Diaz-Curiel M, Harvey N et al (2013) Standardising the descriptive epidemiology of osteoporosis: recommendations from the epidemiology and quality of life working group of IOF. Osteoporos Int 24:2763–2764. 10.1007/s00198-013-2413-7 PMC5096926 23884436

[27] Fernández PC, Castro CMJ, Sánchez NL, Rapún ML, Morales IC, Arrobas VT (2023) Handling of lipemic samples in the clinical laboratory. Adv Lab Med 4:5–27. 10.1515/almed-2023-0003 PMC10197190 37359904

[28] Omair MA, Pagnoux C, McDonald-Blumer H, Johnson SR (2013) Low bone density in systemic sclerosis. A systematic review. J Rheumatol 40:1881–1890. 10.3899/jrheum.130032 24037552

[29] Schwetz V, Trummer C, Pandis M, Grübler MR, Verheyen N, Gaksch M, Zittermann A et al (2017) Effects of vitamin D supplementation on bone turnover markers: a randomized controlled trial. Nutrients 9:432. 10.3390/nu9050432 PMC5452162 28448457

[30] Greenblatt MB, Shim JH (2013) Osteoimmunology: a brief introduction. Immune Netw 13:111–115. 10.4110/in.2013.13.4.111 PMC3759707 24009537

[31] Szekanecz Z, Raterman HG, Petho Z, Lems WF (2019) Common mechanisms and holistic care in atherosclerosis and osteoporosis. Arthritis Res Ther 21:15. 10.1186/s13075-018-1805-7 PMC6329187 30630495

[32] Amarasekara DS, Yu J, Rho J (2015) Bone loss triggered by the cytokine network in inflammatory autoimmune diseases. J Immunol Res 2015:832127. 10.1155/2015/832127 PMC4434203 26065006

[33] Gamal RM, Gamal WM, Ghandour AM, Abozaid HSM, Mohamed ME, Emad Y, Abdel Galeel A (2018) Study of the osteoprotegerin/receptor activator of nuclear factor-kB ligand system association with inflammation and atherosclerosis in systemic sclerosis. Immunol Invest 47:241–250. 10.1080/08820139.2017.1423499 29336616

[34] Li J, Sarosi I, Cattley RC, Pretorius J, Asuncion F, Grisanti M, Morony S et al (2006) Dkk1-mediated inhibition of Wnt signaling in bone results in osteopenia. Bone 39:754–766. 10.1016/j.bone.2006.03.017 16730481

[35] Taylan A, Birlik M, Kenar G, Toprak B, Gundogdu B, Gurler O, Karakas B et al (2019) Osteoprotegrin interacts with biomarkers and cytokines that have roles in osteoporosis, skin fibrosis, and vasculopathy in systemic sclerosis: A potential multifaceted relationship between OPG/RANKL/TRAIL and Wnt inhibitors. Mod Rheumatol 29:619–624. 10.1080/14397595.2018.1500736 30001654

[36] Sung EA, Park MH, Henegariu O, Sime PJ, Chae WJ (2023) Dickkopf1 promotes pulmonary fibrosis upon bleomycin-induced lung injury. Am J Pathol 193:1130–1142. 10.1016/j.ajpath.2023.05.009 PMC10477954 37263344

[37] Ray S, Khassawna T, El, Sommer U, Thormann U, Wijekoon ND, Lips K, Heiss C et al (2017) Differences in expression of Wnt antagonist Dkk1 in healthy versus pathological bone samples. J Microsc 265:111–120. 10.1111/jmi.12469 27580425

[38] Kudlacek S, Schneider B, Woloszczuk W, Pietschmann P, Willvonseder R, Austrian Study Group on Normative Values of Bone Metabolism (2003) Serum levels of osteoprotegerin increase with age in a healthy adult population. Bone 32:681–686. 10.1016/s8756-3282(03)00090-5 12810175

[39] Lv Y, Wang G, Xu W, Tao P, Lv X, Wang Y (2015) Tartrate-resistant acid phosphatase 5b is a marker of osteoclast number and volume in RAW 264.7 cells treated with receptor-activated nuclear κB ligand. Exp Ther Med 9:143–146. 10.3892/etm.2014.2071 PMC4247282 25452790

[40] Soós B, Szentpétery Á, Raterman HG, Lems WF, Bhattoa HP, Szekanecz Z (2022) Effects of targeted therapies on bone in rheumatic and musculoskeletal diseases. Nat Rev Rheumatol 18:249–257. 10.1038/s41584-022-00764-w 35273387

[41] Gulyás K, Horváth Á, Végh E, Pusztai A, Szentpétery Á, Pethö Z, Váncsa A et al (2020) Effects of 1-year anti-TNF-α therapies on bone mineral density and bone biomarkers in rheumatoid arthritis and ankylosing spondylitis. Clin Rheumatol 39:167–175. 10.1007/s10067-019-04771-3 31522318

[42] Pusztai A, Hamar A, Czókolyová M, Gulyás K, Horváth Á, Végh E, Pethő Z et al (2021) Associations of vascular and bone status in arthritis patients. Sci Rep 11:19504. 10.1038/s41598-021-99071-9 PMC8484288 34593938

[43] Szentpétery Á, Horváth Á, Gulyás K, Pethö Z, Bhattoa HP, Szántó S, Szücs G et al (2017) Effects of targeted therapies on the bone in arthritides. Autoimmun Rev 16:313–320. 10.1016/j.autrev.2017.01.014 28159704

[44] Juhász B, Gulyás K, Horváth Á, Végh E, Pusztai A, Szentpétery Á, Pethő Z et al (2021) Peripheral quantitative computed tomography in the assessment of bone mineral density in anti-TNF-treated rheumatoid arthritis and ankylosing spondylitis patients. BMC Musculoskelet Disord 22:817. 10.1186/s12891-021-04708-5 PMC8461899 34556105

[45] Pethő Z, Kalina E, Pap Z, Hodosi K, Falcsik R, Balogh Á, Szekanecz Z et al (2021) Characterization of bone metabolism in hungarian psoriatic arthritis patients: a case–control study. BMC Musculoskelet Disord 22:70. 10.1186/s12891-021-03952-z PMC7805054 33435899

