# Supplementary material for: Compartment-specific skeletal involvement in systemic sclerosis characterized by peripheral QCT and serum osteoimmune mediators: a case-control study
Source: Calcif Tissue Int. 2026 Oct 22;117(1):166. doi: 10.1007/s00223-026-01624-5 (PMC13690878; doi:10.1007/s00223-026-01624-5)
Supplement: Supplementary file 1 [file 223_2026_1624_MOESM1_ESM.docx]

Compartment-specific skeletal involvement in systemic sclerosis characterized by peripheral QCT and serum osteoimmune mediators: a case-control study _ ESM

Calcified Tissue International

Dóra Csige^1,2*^, Ágnes Horváth^1^, Monika Bodoki^1^, István Rebenku^3^, Sára Lipták-Lukácsik^1^, Ramóna Lilla Pomázi^1^, Zsófia Pethő^1^, Sándor Mogyoróssy^1^, Attila Hamar^1^, Nóra Bodnár^1^, Rebeka Falcsik^1^, Katalin Hodosi^1^, György Panyi^3^, Szilvia Szamosi^1^, Zoltán Szekanecz^1^, Gabriella Szűcs^1^, Edit Végh^1^# and Levente Bodoki^1,2^#

^1^ Division of Rheumatology, Department of Internal Medicine, Faculty of Medicine, University of Debrecen, Debrecen, Hungary

^2^ Doctoral School of Clinical Medicine, University of Debrecen, Debrecen, Hungary

^3^ Department of Biophysics and Cell Biology, Faculty of Medicine, University of Debrecen, Debrecen, Hungary

#These authors contributed equally to this work.

*Corresponding author: Dóra Csige

Móricz Zsigmond út 22., H-4032, Debrecen, Hungary

e-mail address: csige.dora@med.unideb.hu

phone: +36705111889

**SI1. Flow diagram of participant inclusion and data availability.** The study included 40 women with systemic sclerosis (SSc) and 45 non-SSc controls. DXA measurements, FRAX estimates, and routine laboratory data were available for all participants. pQCT measurements were available for 31 of 40 patients with SSc and 35 of 45 controls; unavailable pQCT data were attributable to unavailable scans, motion artefacts, or measurements not being performed. Osteoimmune biomarker data were available for 38 patients with SSc and all 45 controls; two SSc serum samples were excluded from biomarker analyses because of gross lipemia.

**SI2 Data availability across study assessments**

| **Assessment** | **SSc available/total (%)** | **Non-SSc available/total (%)** |
| --- | --- | --- |
| DXA | 40/40 (100%) | 45/45 (100%) |
| pQCT | 31/40 (77.5%) | 35/45 (77.8%) |
| FRAX | 40/40 (100%) | 45/45 (100%) |
| Routine laboratory parameters | 40/40 (100%) | 45/45 (100%) |
| Osteoimmune biomarkers | 38/40 (95.0%) | 45/45 (100%) |

Data are presented as the number of participants with available data/total number of participants (%). Missing pQCT data were attributable to unavailable scans, motion artefacts, or measurements not being performed. Osteoimmune biomarker data were unavailable for two patients with SSc because the serum samples were unsuitable for analysis due to gross lipemia.

**SI3 Routine laboratory markers of bone and mineral metabolism**

| **Marker** | **Reference range** | **SSc mean ± SD** | **SSc outside ref. (low/high)** | **Non-SSc mean ± SD** | **Non-SSc outside ref. (low/high)** | **p-value** |
| --- | --- | --- | --- | --- | --- | --- |
| Total calcium (mmol/L) | 2.1–2.6 | 2.37 ± 0.11 | high: 1 (2.5%) | 2.38 ± 0.09 | high: 1 (2.2%) | 0.761 |
| Phosphate (mmol/L) | 0.8–1.45 | 1.09 ± 0.14 | 0 | 1.16 ± 0.32 | high: 1 (2.2%) | 0.382 |
| Vitamin B12 (pmol/L) | 145–637 | 358.99 ± 132.92 | low: 1 (2.5%)  high: 2 (5.0%) | 334.35 ± 114.96 | low: 1 (2.2%) | 0.632 |
| Osteocalcin (µg/L) | ≤41 | 16.23 ± 7.67 | 0 | 19.50 ± 7.26 | 0 | **0.029** |
| β-CTX (µg/L) | ≤0.573 | 0.31 ± 0.18 | high: 4 (10.0%) | 0.27 ± 0.16 | high: 2 (4.4%) | 0.231 |
| P1NP (µg/L) | ≤75 | 46.89 ± 19.13 | high: 3 (7.5%) | 46.70 ± 22.31 | high: 6 (13.3%) | 0.529 |
| Bone-specific ALP (µg/L) | 5–25 | 13.05 ± 4.26 | low: 4 (10.0%)  high: 3 (7.5%) | 11.19 ± 5.22 | low: 3 (6.7%)  high: 1 (2.2%) | 0.909 |
| PTH (pmol/L) | 1.6–6.9 | 3.78 ± 1.40 | high: 2 (5.0%) | 4.08 ± 1.32 | high: 1 (2.2%) | 0.203 |
| 25(OH)D (nmol/L) | ≥75 | 92.65 ± 28.80 | low: 10 (25.0%) | 83.16 ± 28.79 | low: 18 (40.0%) | **0.031** |
| Folate (nmol/L) | 10.4–42.4 | 18.05 ± 9.12 | low: 6 (15.0%)  high: 6 (15.0%) | 16.37 ± 7.22 | low: 8 (17.8%)  high: 3 (6.7%) | 0.173 |

Abbreviations: SSc: systemic sclerosis; ALP: alkaline phosphatase; β-CTX: C-terminal telopeptide of type I collagen; P1NP: procollagen type I N-terminal propeptide; PTH: parathyroid hormone; 25(OH)D: 25-hydroxyvitamin D

Footnote: Data are presented as mean ± standard deviation (SD) unless otherwise indicated. “Outside reference range” denotes the number and percentage of participants with values below (low) or above (high) the laboratory-specific reference limits. Between-group comparisons were performed using parametric or non-parametric tests as appropriate, based on data distribution. P values refer to comparisons between SSc patients and non-SSc controls. Reference ranges correspond to those used by the local certified clinical laboratory.

**SI4 Baseline demographic, clinical and bone related characteristics of SSc subtypes**

| **Parameters** | | | **lcSSc (n=21)** | **dcSSc (n=19)** | **p-value** |
| --- | --- | --- | --- | --- | --- |
| Age, years, mean ± SD | | | 55.90 ± 9.14 | 58.68 ± 10.20 | 0.369 |
| BMI, mean ± SD | | | 25.95 ± 4.27 | 26.30 ± 4.97 | 0.578 |
| Bone status groups | osteoporosis, n (%) | | 5 (24.0) | 7 (37.0) | 0.494 |
|  | osteopenia, n (%) | | 10 (48.0) | 8 (42.0) | 0.760 |
|  | normal bone, n (%) | | 6 (29.0) | 4 (21.0) | 0.583 |
| SSc duration (months), mean ± SD | | | 49.5 ± 27.24 | 42.6 ± 22.03 | 0.498 |
| Age at SSc diagnosis (years), mean ± SD | | | 54.63 ± 10.51 | 52.19 ± 9.45 | 0.444 |
| Patients in menopause, n (%) | | | 16 (76.2) | 15 (78.9) | 0.834 |
| Menopausal age (years), mean ± SD | | | 47.56 ± 3.91 | 46.29 ± 7.19 | 0.807 |
| Duration of menopause (years), mean ± SD | | | 14.13 ± 7.74 | 13.33 ± 9.57 | 0.801 |
| Current smoking, n (%) | | | 1 (4.8) | 2 (10.6) | 0.489 |
| Alcohol intake (occasionally), n (%) | | | 4 (32.5) | 8 (37.8) | 0.112 |
| Treatments, n (%) | Corticosteroid ( > 5mg methylprednisolone) | | 0 (0) | 0 (0) | - |
|  | MMF (current) | | 16 (76.2) | 8 (42.1) | 0.051 |
|  | Tocilizumab (current) | | 1 (4.8) | 5 (26.3) | 0.056 |
|  | Cyclophosphamide (former) | | 4 (32.5) | 7 (36.8) | 0.208 |
|  | Rituximab (current) | | 0 (0) | 4 (21.1) | 0.012 |
|  | Bisphosphonates (current) | | 2 (9.5) | 2 (10.5) | 0.916 |
|  | Denosumab (current) | | 2 (9.5) | 1 (5.26) | 0.609 |
|  | D-vitamin supplementation | | 21 (100.0) | 19 (100.0) | 0.999 |
|  | Calcium supplementation | | 7 (33.3) | 6 (31.6) | 0.999 |
| UCLA GIT 2.0 total score, median (Q1-Q3) | | | 0.22 (0.08-0.47) | 0.12 (0.00-0.30) | 0.275 |
| FRAX major osteoporotic %, median (Q1-Q3) | | | 8.81 (1.1-21.2) | 13.24 (0.8-67.0) | 0.440 |
| FRAX hip %, median (Q1-Q3) | | | 3.30 (0.1-13.2) | 7.56 (0.1-65.0) | 0.455 |
| DXA BMD (g/cm^2^) | | L1–4, mean ± SD | 1.13 ± 0.18 | 1.13 ± 0.24 | 0.981 |
|  |  | FN, mean ± SD | 0.84 ± 0.12 | 0.84 ± 0.19 | 0.963 |
| DXA T-score | | L1–4, mean ± SD | -0.47 ± 1.43 | -0.52 ± 1.91 | 0.926 |
|  |  | FN, mean ± SD | -1.21 ± 1.00 | -1.23 ± 1.56 | 0.977 |
| **Parameters** | | | **lcSSc (n=18)** | **dcSSc (n=13)** | **p-value** |
| pQCT vBMD (mg/cm^3^); mean ± SD | | Radius total | 299.57 ± 73.65 | 286.81 ± 79.73 | 0.626 |
|  |  | Radius trabecular | 152.19 ± 41.51 | 149.39 ± 45.47 | 0.850 |
|  |  | Radius cortical+subcortical | 419.83 ± 113.75 | 398.88 ± 123.66 | 0.605 |
| pQCT T-score | | Radius total | -1.44 ± 1.23 | -1.66 ± 1.34 | 0.626 |
|  |  | Radius trabecular | -1.24 ± 1.03 | -1.31 ± 1.14 | 0.868 |
| pQCT Z-score | | Radius total | -0.71 ± 0.90 | -0.74 ± 1.34 | 0.950 |
|  |  | Radius trabecular | -0.84 ± 1.01 | -0.90 ± 0.92 | 0.859 |
| **Parameters** | | | **lcSSc (n=21)** | **dcSSc (n=19)** | **p-value** |
| Total calcium (mmol/L), mean ± SD | | | 2.38 ± 0.10 | 2.35 ± 0.12 | 0.436 |
| Phosphate (mmol/L), mean ± SD | | | 1.07 ± 0.15 | 1.13 ± 0.11 | 0.163 |
| Vitamin B12 (pmol/L), mean ± SD | | | 350.68 ± 137.02 | 366.42 ± 132.45 | 0.196 |
| Osteocalcin (µg/L), mean ± SD | | | 14.56 ± 6.88 | 18.08 ± 8.26 | 0.149 |
| β-CTX (µg/L), mean ± SD | | | 0.29 ± 0.16 | 0.35 ± 0.19 | 0.291 |
| P1NP (µg/L), mean ± SD | | | 42.43 ± 16.84 | 51.82 ± 20.72 | 0.123 |
| Bone-specific ALP (µg/L), mean ± SD | | | 14.21 ± 18.78 | 11.77 ± 6.73 | 0.596 |
| PTH (pmol/L), mean ± SD | | | 3.64 ± 1.43 | 3.93 ± 1.39 | 0.527 |
| 25(OH)D (nmol/L), mean ± SD | | | 88.77 ± 29.11 | 96.95 ± 28.60 | 0.377 |
| Folate (nmol/L), mean ± SD | | | 19.80 ± 9.55 | 18.11 ± 10.76 | **0.031** |
| **Parameters** | | | **lcSSc (n=20)** | **dcSSc (n=18)** | **p-value** |
| OPG (pg/ml), mean ± SD | | | 2288.78 ± 1031.69 | 2203.59 ± 752.56 | 0.769 |
| OPN (pg/ml), mean ± SD | | | 6369.80 ± 5304.11 | 7963.01 ± 4305.71 | 0.307 |
| PDGF-BB (pg/ml), mean ± SD | | | 22483.1 ± 14690.8 | 24013.3 ± 14580.4 | 0.743 |
| ACP5 (pg/ml), mean ± SD | | | 7397.73 ± 2441.26 | 7364.09 ± 2587.35 | 0.810 |
| RANKL (pg/ml), mean ± SD | | | 443.37 ± 46.83 | 564.42 ± 37.72 | 0.260 |
| TNF-α (pg/ml), mean ± SD | | | 35.18 ± 31.57 | 36.79 ± 31.97 | 0.535 |
| IL-6 (pg/ml), mean ± SD | | | 76.38 ± 50.09 | 137.96 ± 126.95 | **0.003** |
| IL-1β (pg/ml), mean ± SD | | | 25.47 ± 7.45 | 23.41 ± 5.28 | 0.131 |
| DKK-1 (pg/ml), mean ± SD | | | 996.08 ± 344.30 | 1004.34 ± 378.77 | 0.544 |
| RANKL/OPG, mean ± SD | | | 0.19 ± 0.2 | 0.30 ± 0.4 | 0.300 |

Abbreviations: SSc: systemic sclerosis; lcSSc: limited cutaneous systemic sclerosis; dcSSc: diffuse cutaneous systemic sclerosis; BMI: body mass index; DXA: dual-energy X-ray absorptiometry; BMD: bone mineral density; FN: femoral neck; OP: osteoporosis; OPe: osteopenia; FRAX: Fracture Risk Assessment Tool; UCLA GIT 2.0: University of California Los Angeles Scleroderma Clinical Trial Consortium Gastrointestinal Tract Instrument 2.0; MMF: mycophenolate mofetil; pQCT: peripheral quantitative computed tomography; SD: standard deviation; ALP: alkaline phosphatase; β-CTX: C-terminal telopeptide of type I collagen; P1NP: procollagen type I N-terminal propeptide; PTH: parathyroid hormone; 25(OH)D: 25-hydroxyvitamin D; ACP5: acid phosphatase 5 (tartrate-resistant acid phosphatase 5b); DKK-1: Dickkopf-related protein 1; IL: interleukin; OPG: osteoprotegerin; OPN: osteopontin; PDGF-BB: platelet-derived growth factor-BB; RANKL: receptor activator of nuclear factor κB ligand; TNF-α: tumor necrosis factor alpha.

Footnote: Values are presented as mean ± standard deviation (SD), median (interquartile range, Q1–Q3), or number (percentage), as appropriate. Bone status categories were defined according to DXA-based T-score criteria. P values were calculated using independent-samples t test or Mann–Whitney U test for continuous variables and χ² or Fisher’s exact test for categorical variables, as appropriate. A two-sided p value <0.05 was considered statistically significant. Sample sizes vary across assessment domains according to data availability, as indicated in the table headings.

**SI5 Correlation between chronological age and circulating OPG levels in patients with SSc.** Scatter plot showing the association between chronological age and serum osteoprotegerin (OPG) levels in patients with systemic sclerosis (SSc). The correlation coefficient and corresponding p value are shown in the figure.
